# SAFETY AND EFFECTIVENESS OF BLUE EYE HYPERTONIC SOLUTION VERSUS SALINE SOLUTION IN THE TREATMENT OF FLAT COLONIC LESIONS: A RANDOMIZED CLINICAL TRIAL

**DOI:** 10.1590/S0004-2803.24612025-115

**Published:** 2026-05-25

**Authors:** Caio Colturato COIMBRA, Isabela Franzon Leopize DA GAMA, Roberto Luiz KAISER, Fernando Tadeu Vannucci COIMBRA, Carolina Colombelli PACCA, Luiz Gustavo de QUADROS

**Affiliations:** 1 Faculdade de Medicina de São José do Rio Preto (FAMERP), São José do Rio Preto, SP, Brasil.; 2 Kaiser Hospital Dia, São José do Rio Preto, SP, Brasil.; 3 Universidade Estadual Paulista (UNESP), São José do Rio Preto, SP, Brasil.

**Keywords:** Endoscopic mucosal resection, colorectal cancer, Blue Eye, submucosal injection, histopathology, viscoelastic solution, Ressecção endoscópica da mucosa, câncer colorretal, olho azul, injeção submucosa, histopatologia, solução viscoelástica

## Abstract

**Background:**

Colorectal cancer (CRC) represents a significant global health challenge, with early detection and minimally invasive treatments playing key roles in reducing morbidity and mortality. Endoscopic mucosal resection (EMR) is widely employed for flat colonic lesions, and the quality of submucosal elevation is a decisive factor for achieving complete resections, minimizing complications, and ensuring accurate histopathological assessment. Conventional 0.9% saline with dye is the standard solution but dissipates rapidly, while viscoelastic agents such as Blue Eye may offer more durable and technically advantageous lifts.

**Objectives:**

This study aimed to compare the technical and histopathological outcomes of Blue Eye versus 0.9% saline with indigo carmine in EMR of flat colonic lesions, focusing on lateral margins, injection volume, procedural performance, and clinical safety.

**Methods:**

A prospective, single-center, triple-blind study was conducted between January and October 2024, including 14 patients undergoing 19 EMRs for Paris 0-IIa flat colonic lesions sized 20-30 mm. Patients were randomized to receive either Blue Eye or saline with indigo carmine. Baseline demographics, comorbidities, lesion features, procedural variables, and histopathological outcomes were collected. Statistical analysis included the Mann-Whitney test for continuous variables and Fisher’s exact test for categorical data, adopting *P*<0.05 as significant.

**Results:**

Baseline characteristics were comparable between groups regarding age, sex, and comorbidities. Blue Eye demonstrated significantly larger lateral margins (2.61±1.19 mm vs 1.81±0.37 mm, *P*=0.047) and required lower injection volume (4.60±1.50 mL vs 10.00±7.21 mL, *P*=0.020). Other variables, including procedure time, en bloc resection rate, bleeding, and number of clips, did not differ significantly. Anatomical lesion distribution and unfavorable anatomical findings also showed no statistical association with the solution employed.

**Conclusion:**

Blue Eye appears to provide superior technical performance in EMR of flat colonic lesions by ensuring wider lateral margins with less injection volume, without increasing adverse outcomes. Larger multicenter studies are needed to confirm and generalize these findings.

## Abstract (key points)

• Blue Eye solution provided significantly wider lateral margins compared to saline with indigo carmine in EMR of flat colonic lesions.

• Lower injection volumes were required with Blue Eye, reducing procedural manipulation.

• Clinical and safety outcomes were similar between groups, with no increase in adverse events.

• Blue Eye demonstrates potential as a superior submucosal injectate, warranting validation in larger multicenter trials.

## RESUMO

**Contexto:**

O câncer colorretal (CCR) representa um desafio significativo para a saúde global, com a detecção precoce e os tratamentos minimamente invasivos desempenhando papéis fundamentais na redução da morbidade e mortalidade. A ressecção endoscópica da mucosa (RME) é amplamente empregada para lesões colônicas planas, e a qualidade da elevação da submucosa é um fator decisivo para alcançar ressecções completas, minimizar complicações e garantir uma avaliação histopatológica precisa. A solução salina convencional a 0,9% com corante é a solução padrão, mas se dissipa rapidamente, enquanto agentes viscoelásticos como o Blue Eye podem oferecer elevações mais duráveis e tecnicamente vantajosas.

**Objetivo:**

Este estudo teve como objetivo comparar os resultados técnicos e histopatológicos do Blue Eye versus solução salina a 0,9% com índigo carmim na RME de lesões colônicas planas, com foco nas margens laterais, volume de injeção, desempenho do procedimento e segurança clínica.

**Métodos:**

Um estudo prospectivo, unicêntrico e triplo-cego foi conduzido entre janeiro e outubro de 2024, incluindo 14 pacientes submetidos a 19 EMRs para lesões colônicas planas Paris 0-IIa medindo 20-30 mm. Os pacientes foram randomizados para receber Blue Eye ou solução salina com índigo carmim. Dados demográficos basais, comorbidades, características da lesão, variáveis do procedimento e desfechos histopatológicos foram coletados. A análise estatística incluiu o teste de Mann-Whitney para variáveis contínuas e o teste exato de Fisher para dados categóricos, adotando p < 0,05 como significativo.

**Resultados:**

As características basais foram comparáveis entre os grupos em relação à idade, sexo e comorbidades. Blue Eye demonstrou margens laterais significativamente maiores (2,61±1,19 mm vs 1,81±0,37 mm, *P*=0,047) e exigiu menor volume de injeção (4,60±1,50 mL vs 10,00±7,21 mL, *P*=0,020). Outras variáveis, incluindo tempo de procedimento, taxa de ressecção em bloco, sangramento e número de clipes, não diferiram significativamente. A distribuição anatômica da lesão e os achados anatômicos desfavoráveis também não mostraram associação estatística com a solução empregada.

**Conclusão:**

Blue Eye parece fornecer desempenho técnico superior em EMR de lesões colônicas planas, garantindo margens laterais mais amplas com menos volume de injeção, sem aumentar os resultados adversos. Estudos multicêntricos maiores são necessários para confirmar e generalizar esses achados.

## INTRODUCTION

Colorectal cancer (CRC) remains a major public-health burden, driven by a multifactorial etiology that spans genetic predisposition and modifiable exposures^1^ ^,^ ^2^. The expansion of colonoscopic screening and surveillance has increased the detection of early neoplastic lesions^3^, particularly lateral-spreading lesions (Paris 0-II) that are amenable to endoscopic mucosal resection (EMR)^4^ ^,^ ^5^. EMR hinges on reliable submucosal lifting to create a safe dissection plane, a step that directly influences en bloc resection rates, adverse events, and, critically, the histopathological assessment of lateral and deep margins that guides prognosis and subsequent management^6^ ^,^ ^7^.

Despite its centrality, the submucosal injection phase lacks standardization, with solutions differing in viscosity, durability of cushion, tissue staining, and cost^8^. The conventional 0.9% saline with dye is inexpensive and widely available but dissipates rapidly, often necessitating reinjections and potentially compromising lift quality, procedure efficiency, and margin adequacy^8^ ^,^ ^9^. More viscoelastic agents-such as hyaluronic-acid-based preparations-may sustain elevation longer and facilitate controlled resection, yet comparative evidence in flat colonic lesions remains limited and heterogeneous, and histopathology-focused endpoints are underreported. This uncertainty hampers evidence-based selection of injectates and may translate into variable technical performance and specimen quality across centers^10^ ^,^ ^11^.

Despite its centrality, the submucosal injection phase lacks standardization, with solutions differing in viscosity, durability of cushion, tissue staining, and cost^8^. The conventional 0.9% saline with dye is inexpensive and widely available but dissipates rapidly, often necessitating reinjections and potentially compromising lift quality, procedure efficiency, and margin adequacy^8^ ^,^ ^9^. In this context, viscoelastic agents have emerged as promising alternatives. Blue Eye (GI Supply, Inc., Camp Hill, PA) is a commercially available, pre-mixed solution composed of sodium hyaluronate, sodium chloride, and a biocompatible blue dye. Its mechanism of action relies on the high viscosity of hyaluronic acid, which creates a more stable and durable submucosal cushion compared to saline. Previous studies have suggested that such agents may sustain elevation longer and facilitate controlled resection, yet comparative evidence in flat colonic lesions remains limited and heterogeneous, with a particular scarcity of data on histopathology-focused endpoints^10^ ^,^ ^11^. While the higher cost of viscoelastic solutions compared to saline is a relevant consideration for clinical adoption, a comprehensive evaluation must also account for potential benefits in procedural efficiency, specimen quality, and reduced complication rates.

This study seeks to address the limitations of conventional submucosal injection solutions by exploring agents that can enhance both technical performance and histopathological reliability in EMR. By investigating the comparative performance of Blue Eye against the standard saline-dye approach, this research aims to generate evidence that may guide the adoption of more effective injectates in clinical practice. The objective of this research is to compare the efficacy of Blue Eye versus saline with indigo carmine in submucosal elevation for colonic EMR, with a focus on technical, histological, and clinical parameters that can inform safer and more standardized endoscopic oncology care.

## METHODS

### Study design

This was a single-center, prospective, cross-sectional, triple-blind clinical investigation with a quantitative approach, conducted in a specialized digestive endoscopy unit. Eligible participants were individuals undergoing elective colonoscopy in whom flat colonic lesions, classified as Paris 0-IIa and measuring between 20 and 30 mm, were identified. The study employed an analytical and descriptive framework to compare two injectable solutions used in endoscopic mucosal resection (EMR).

### Setting and period

The research was carried out at Kaiser Clinic, located in São José do Rio Preto, São Paulo, Brazil, between January and October 2024, with systematic monitoring of all procedures and subsequent histopathological analyses.

### Inclusion criteria and exclusion criteria

Patients aged 18 years or older, of either sex, presenting with flat colonic lesions type 0-IIa measuring 20-30 mm, and scheduled for elective colonoscopy with EMR were considered eligible, provided they signed the informed consent form. Exclusion criteria comprised inadequate bowel preparation, lesions without adequate submucosal lifting following injection, specimens unsuitable for histopathological evaluation, and incomplete resections.

### Procedures

Participants were randomly assigned to one of two groups: a control group receiving 0.9% saline with indigo carmine or an intervention group receiving Blue Eye solution. Randomization was performed by an endoscopy nurse using a computerized system. To ensure blinding of patients, endoscopists, and pathologists, the solutions were prepared in identical 5 ml syringes. The control solution consisted of saline with indigo carmine in a concentration adjusted to achieve a color similar to that of Blue Eye. The Blue Eye content was transferred to an identical 5 ml syringe. EMR was carried out following standardized technical protocols. Chromoendoscopy with indigo carmine was performed to better delineate lesion borders prior to resection. All resected specimens were analyzed by an experienced pathologist blinded to the solution used.

### Variables analyzed

Collected data included demographic characteristics (age, sex), clinical information (hypertension, diabetes, smoking status), lesion features (size, location, Paris classification, histological type), technical parameters (procedure time, injected volume, complications, number of fragments), and histopathological outcomes. For histopathological assessment, specimens were fixed in formalin, sectioned, and stained with hematoxylin and eosin. Lateral margins were measured in millimeters (mm) from the edge of the neoplastic tissue to the thermal cautery artifact at the specimen’s edge. Lesion depth was assessed by microscopic examination to determine the extent of invasion into the submucosa or deeper layers.

### Histopathological assessment

Resected specimens were fixed in 10% formalin and submitted for standard histopathological analysis. In the pathology laboratory, each specimen was oriented, stained, and subjected to serial sectioning for evaluation of resection margins. Lateral margins were measured in millimeters, with the study considering the shortest distance between the tumor edge and any lateral margin (‘minimum margin’). The deep margin was also measured in millimeters, corresponding to the distance between the base of the lesion and the resection plane. All measurements were performed under optical microscopy by experienced pathologists, following conventional histological criteria for mucosal resection.

### Statistical analysis

The statistical unit of analysis was the resected lesion (n=19), not the patient (n=14). Baseline homogeneity between groups was assessed not only for patient demographics (age, sex) but also for lesion characteristics (size and location). Normality was assessed with the Shapiro-Wilk test, revealing that several quantitative variables did not follow a normal distribution. Consequently, nonparametric methods were employed. Group comparisons for numerical variables were performed using the Mann-Whitney test, while categorical variables were compared using Fisher’s exact test. Statistical analyses were conducted with SPSS (Statistical Package for the Social Sciences), adopting a significance level of *P*<0.05.

### Ethical considerations

The study protocol was reviewed and approved by the Research Ethics Committee of Kaiser Clinic, in accordance with Resolution CNS no. 466/2012. All participants provided written informed consent, and their rights to autonomy, confidentiality, and voluntary withdrawal from the study at any stage were fully respected without compromising medical care.

## RESULTS

A total of 14 patients underwent 19 endoscopic mucosectomy procedures. The lesions were randomized into two groups: 10 lesions (52.6%) were resected using Blue Eye solution, and 9 lesions (47.4%) were resected using saline solution with indigo carmine. Table 1 summarizes the demographic and clinical characteristics of the study population. All individuals were over 18 years of age, comprising both male and female participants, with lesions classified as flat type and measuring between 20 and 30 mm in diameter. The age distribution indicated a mean of 65.64 years, with a median of 64 years and a range from 42 to 88 years. In addition, the table provides an overview of the frequency and proportion of comorbidities and risk factors identified in the sample.

**TABLE 1:** Demographic and clinical characteristics of patients undergoing endoscopic mucosectomy.

Total (n=14 patients / 19 procedures)	Baseline Characteristics	Frequency (n)	Percentage (%)
Sex	Male	6	42.9
	Female	8	57.1
Age (years)	Mean ± SD	65.64 ± 12.30	-
	Median (IQR)	64 (54-73)	-
	Range	42-88	-
Lesion type	Flat (Paris 0-IIa)	19 procedures	100.0
Lesion size	20-30 mm	19 procedures	100.0
Clinical comorbidity	Diabetes Mellitus	4	28.6
	Hypertension	7	50.0
	Smoking	2	14.3

Table 2 presents the comparative analysis between the two study groups regarding key variables associated with endoscopic mucosectomy procedures. The variables evaluated included both demographic characteristics and technical aspects of the intervention, such as lateral margins, procedure time, injection volume, resection type, number of clips applied, and intraoperative bleeding. Overall, the groups demonstrated homogeneity with respect to age distribution (*P*=0.211) and sex (*P*=0.650), suggesting that the baseline demographic profiles were well balanced. Statistically significant differences were identified for two procedural variables. Lateral margins were significantly larger in the Blue Eye group compared with the Physiological Saline with Indigo Carmine group (*P*=0.047), with a mean difference of 0.8 mm. Similarly, the injection volume was significantly greater in the Indigo Carmine group (*P*=0.020), with an average difference of 5.4 mL between groups. Other outcomes, including procedure time (*P*=0.380), number of clips (*P*=0.310), type of resection (*P*=0.212), and bleeding occurrence (*P*=0.417), did not reach statistical significance.

**TABLE 2:** Comparison between study groups based on variables related to endoscopic mucosectomy procedures.

Variable		Blue Eye (n=10, 52.6%)	Physiological Saline with Indigo Carmine (n=9, 47.4%)	***P*-value**
Age (years)		62.00 ± 13.85	69.33 ± 7.00	0.211^a^
Sex	Male, n (%)	3 (30.0)	4 (44.4)	0.650^b^
	Female, n (%)	7 (70.0)	5 (55.6)	
Lateral margins (mm)		2.61 ± 1.19	1.81 ± 0.37	0.047^a^
Procedure time (minutes)		7.00 ± 2.40	9.67 ± 5.72	0.380^a^
Injection volume (ml)		4.60 ± 1.50	10.00 ± 7.21	0.020^a^
Resection	En bloc, n (%)	9 (90.0)	6 (66.7)	0.212^b^
	Piecemeal, n (%)	1 (10.0)	3 (33.3)	
Number of clips		1.90 ± 1.11	1.56 ± 1.42	0.310^a^

Notes: values are expressed as mean ± standard deviation or n (%). ^a^Student’s t-test; ^b^Chi-square test. Bold values indicate statistical significance (*P*<0.05).

**FIGURE 1:**
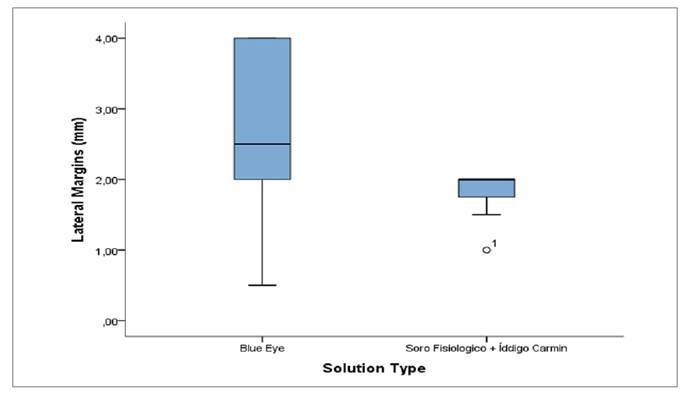
Lateral Margins (mm) according to the type of solution used.

The boxplot in Figure 1 reveals a visible difference in the distribution of lateral margins between the two groups. In the Blue Eye group, greater variability was observed, with a wider interquartile range. The median was approximately 2.5 mm, and values ranged from about 0.5 mm to 4.0 mm, suggesting potentially larger margins with this solution. On the other hand, in the Physiological Saline + Indigo Carmine group, variability was smaller (about 0.38 mm), and the median was close to 2 mm. In addition, an outlier below 1 mm was identified, marked with a small circle, which may indicate a tendency toward more uniform and smaller margins with this solution.

**FIGURE 2:**
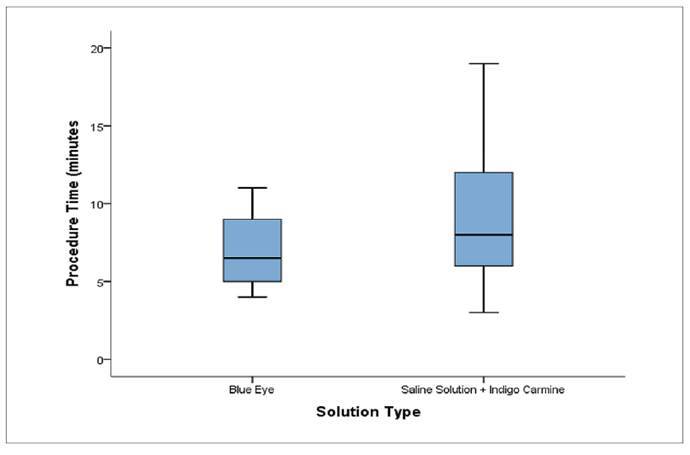
Intervention Time (minutes) according to the type of solution.

The comparison of procedure time between the two solutions revealed differences in data dispersion (Figure 2). The Blue Eye solution showed less variability, with times ranging between 4 and 11 minutes and a median of approximately 7 minutes. In contrast, the Physiological Saline with Indigo Carmine solution showed greater variability, with times ranging from 3 to 19 minutes and a slightly higher median, also around 7 minutes. Despite similar medians, a wider interquartile range was observed in the Physiological Saline with Indigo Carmine group, suggesting greater heterogeneity in procedure times.

**FIGURE 3:**
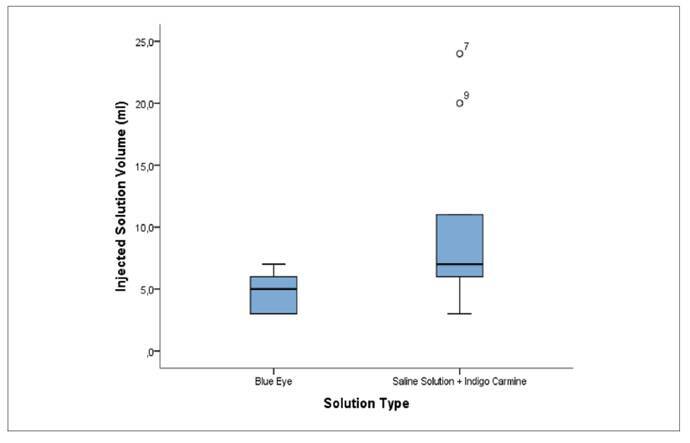
Injection solution volume (ml) by intervention group.

The comparison of injection volumes between the two solutions revealed differences in data dispersion (Figure 3). The Blue Eye solution showed less variability, with volumes distributed between approximately 3 and 7 mL and a median of 5 mL. In contrast, the Physiological Saline with Indigo Carmine solution showed greater variability, with volumes ranging from about 3 to 14 mL and a slightly higher median, close to 7 mL. Despite similar medians, a wider interquartile range was observed in the Physiological Saline with Indigo Carmine group, along with two outliers above 20 mL, suggesting greater heterogeneity in the volume required for submucosal elevation in this group.

**FIGURE 4:**
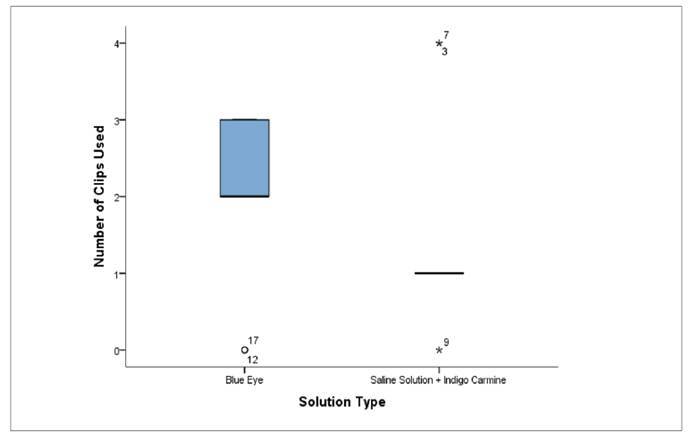
Number of clips used according to the type of solution.

The comparison of the number of clips used between the two solutions revealed differences in both central tendency and data dispersion (Figure 4). The Blue Eye solution showed greater variability, with values distributed between 0 and 3 clips and a higher central tendency compared to the other group (median of 2). The Physiological Saline with Indigo Carmine solution showed a more concentrated distribution, with values ranging between 0 and 4. Although both upper and lower outliers were present, the median in this group was lower than that of the Blue Eye group, indicating a reduced need for clips.

The analysis of lesion profiles subjected to endoscopic mucosectomy revealed that the anatomical location varied between groups, although no significant association was found with the type of solution used for submucosal elevation (*P*=0.910). The mean depth of lesions was 2.4±1.9 mm in the Blue Eye group and 1.5±1.0 mm in the Physiological Saline plus Indigo Carmine group, without statistically significant difference (*P*=0.250). Similarly, the mean lesion size was comparable between groups, measuring 21.5±2.4 mm for Blue Eye and 23.3±4.3 mm for Physiological Saline with Indigo Carmine (*P*=0.360), indicating homogeneity in lesion extent. Table 3 presents the anatomical distribution of lesions according to the solution used, highlighting that lesions located in the cecum were exclusively elevated with Physiological Saline and Indigo Carmine (11.1%), while 55.5% of ascending colon lesions were elevated with this solution compared to 20.0% with Blue Eye. Conversely, lesions in the mid-transverse colon were elevated exclusively with Blue Eye (10%), whereas in the descending colon both solutions were used equivalently (22.2%). In the sigmoid region, a similar distribution was observed, with Blue Eye used in 10.0% of cases and Physiological Saline with Indigo Carmine in 11.1%.

**TABLE 3:** Distribution of lesion location in the colon according to the type of solution used in endoscopic mucosectomy.

Colon segment	Blue Eye n (%)	Physiological Saline + Indigo Carmine n (%)
Cecum	0 (0.0)	1 (11.1)
Ascending colon	2 (20.0)	5 (55.5)
Mid-transverse colon	1 (10.0)	0 (0.0)
Descending colon	2 (22.2)	2 (22.2)
Sigmoid colon	1 (10.0)	1 (11.1)

A statistical analysis was performed to assess whether the frequency of unfavorable anatomical findings differed between the two groups of solutions used during endoscopic mucosectomy (Blue Eye versus Physiological Saline with Indigo Carmine). Given the small sample size and the low frequency observed in several categories, with 75% of the cells presenting an expected count below five, Fisher’s exact test was considered the most appropriate method for this comparison. Table 4 presents the distribution of frequencies according to the type of solution used and the presence of unfavorable anatomical findings. The result (*P*=0.582) indicated no statistically significant association between the solution employed and the occurrence of such findings.

**TABLE 4:** Unfavorable anatomical findings according to the type of solution used in endoscopic mucosectomy.

Type of additional difficulty	Blue Eye (N=10)	Physiological Saline + Indigo Carmine (N=9)
Anatomical alterations in the sigmoid (angulation, redundancy, diverticular disease, splenic angle, hepatic angle, tortuosity)	9 (90.0%)	6 (66.6%)
Fibrosis due to previous tattooing with India ink / lesion in angulation of the transverse colon with partial lifting	1 (10.0%)	1 (11.1%)
Lesions in angulation of the ascending colon	0 (0.0%)	1 (11.1%)
Lesion over diverticular ostium	0 (0.0%)	1 (11.1%)

The histopathological analysis of the 19 resected lesions revealed that all were adenomas. The most common type was tubular adenoma, followed by tubulovillous adenoma. All lesions exhibited low-grade dysplasia. Detailed histological data is presented in Table 5.

**TABLE 5:** Histopathological findings of resected lesions**.

Histological Type	Blue Eye (n=10)	Saline + Indigo Carmine (n=9)	Total (N=19)
Adenoma Type (n, %)			
Tubular	7 (70.0%)	6 (66.7%)	13 (68.4%)
Tubulovillous	3 (30.0%)	3 (33.3%)	6 (31.6%)
Dysplasia Grade (n, %)			
Low-Grade	10 (100%)	9 (100%)	19 (100%)

## DISCUSSION

The present study aimed to compare the efficacy of the viscoelastic solution Blue Eye with the traditional 0.9% Physiological Saline solution with Indigo Carmine in endoscopic mucosectomy of flat colonic lesions. The data obtained demonstrate relevant technical advantages associated with the use of Blue Eye, particularly with respect to wider resection margins and the smaller injection volume required for submucosal elevation^12^ ^-^ ^14^.

The statistically significant difference observed in lateral margins (*P*=0.047) suggests that Blue Eye provides more effective submucosal elevation with reduced dispersion, attributable to its viscoelastic properties, allowing for a wider and safer resection. This feature is critical to achieving histologically free lateral margins, which is essential for therapeutic success and reducing recurrence risk^15^ ^,^ ^16^. Another favorable point for Blue Eye was the significantly lower injection volume required (*P*=0.020), which reinforces the efficiency of this product in producing sustained submucosal lifting with smaller fluid amounts. This advantage may translate into less endoscopic manipulation, fewer reinjections, and potentially lower perforation risk^16^, although such complications were not observed in this study.

Although no statistically significant difference was found for procedure time (*P*=0.380), graphical data demonstrated lower variability in the Blue Eye group, which may indicate greater technical predictability. In addition, the higher en bloc resection rate with Blue Eye (90.0% vs 66.7%) is clinically relevant, even though it did not reach statistical significance (*P*=0.212), likely due to the small sample size. Regarding intraoperative bleeding, the trend toward lower occurrence in the Blue Eye group (10.1%) compared to the control group (22.2%) also points to greater safety, although again without statistical significance. Importantly, the demographic and clinical baseline of the study population (Table 1) showed a balanced distribution between groups in terms of sex, age, and comorbidities such as hypertension, diabetes, and smoking. This homogeneity strengthens the comparability of the groups and reinforces the reliability of the differences identified in procedural outcomes^17^ ^-^ ^20^.

Furthermore, Tables 3 and 4 reveal differences in lesion location between groups, with cecal and ascending colon lesions more frequently resected with saline, while mid-transverse lesions appeared exclusively in the Blue Eye group. While lesions in the cecum and ascending colon were more frequently resected using Physiological Saline and Indigo Carmine, lesions located in the mid-transverse colon appeared exclusively in the Blue Eye group. In the descending and sigmoid colon, both solutions were employed with similar frequency. Taken together, these findings suggest that Blue Eye is feasible across different colonic segments, although Indigo Carmine appeared more commonly in resections of the ascending colon^15^ ^,^ ^21^. Although these differences did not reach statistical significance (*P*=0.410), they may still influence clinically relevant outcomes, such as lateral margin adequacy, given that technical difficulty can vary substantially by anatomical segment. This segmental heterogeneity should be acknowledged when interpreting the results and reinforces the importance of conducting stratified analyses in larger studies, where such differences can be more rigorously evaluated.

The evaluation of unfavorable anatomical findings (Table 4) further demonstrated a higher absolute frequency of difficulties associated with sigmoid alterations in the Blue Eye group. However, this result did not reach statistical significance (*P*=0.582) and is more likely attributable to random variation or individual anatomical differences than to the type of solution itself^22^. Overall, the presence of additional technical challenges varied between groups without a consistent pattern of predominance. The graphical boxplot representations (Figures 1-4) complemented the statistical results by illustrating the distribution, variability, and presence of outliers for lateral margins, procedure time, injection volume, and number of clips. These visual findings reinforced the interpretation that Blue Eye tends to yield larger margins with lower solution volume and more homogeneous operative times^16^ ^,^ ^23^ ^,^ ^24^.

When compared with the literature, the findings are consistent with previous studies, some of which included larger samples, demonstrating similar results: lower injection volume, shorter procedural times, higher in bloc resection rates, wider resection margins, and improved technical feasibility^25^ ^,^ ^26^. However, it is noteworthy that Blue Eye is not yet widely accessible due to its high cost^27^, which may limit its adoption in public and private healthcare systems with restricted budgets. While the higher upfront cost per vial is a relevant consideration, a comprehensive cost-effectiveness analysis should factor in potential savings from reduced procedure times, lower recurrence rates due to wider margins, and fewer required vials per procedure. Such an analysis would be valuable to inform clinical adoption decisions and healthcare policy.

Study limitations include the small number of participants, the unicentric nature of the analysis, the absence of sample size calculation, and the lack of medium- or long-term follow-up variables, which restrict the generalizability of the findings. Despite these limitations, the present results add to the growing body of evidence supporting the use of viscoelastic solutions in endoscopic resection procedures, highlighting technical and potentially clinical benefits.

## CONCLUSION

In this randomized, triple-blind study, Blue Eye solution demonstrated promising technical advantages over standard saline with indigo carmine for EMR of flat colonic lesions. The use of Blue Eye was associated with significantly wider lateral resection margins and required less injection volume, suggesting it may contribute to more reliable and efficient procedures. These benefits were achieved without compromising safety, as clinical outcomes were comparable between the groups. While these findings are encouraging, the conclusions are limited by the small sample size and single-center design. Therefore, rather than asserting its superiority, we conclude that Blue Eye is a promising alternative that warrants further validation in larger, multicenter trials to confirm its efficacy and impact on long-term clinical outcomes such as recurrence rates.

## Data Availability

Data-available-upon-request
